# Factors associated with Women’s timing of first antenatal care visit during their last pregnancy: evidence from 2016 Uganda demographic health survey

**DOI:** 10.1186/s12884-022-05167-z

**Published:** 2022-11-10

**Authors:** Moses Festo Towongo, Enock Ngome, Kannan Navaneetham, Gobopamang Letamo

**Affiliations:** Department of Population Studies, Faculty of Social Sciences, University of Botswana, Gaborone, Botswana

**Keywords:** Individual, Household, Community, Antenatal care visits, Uganda

## Abstract

**Background:**

Utilization of antenatal care services (ANC) during pregnancy has been recognized as a major public health intervention to abate maternal morbidity and mortality. Uganda has experienced high levels of maternal morbidity and mortality over the past two decades. This could be partly attributed to the lower proportion of women who initiated their first antenatal care visit during the first trimester of their gestation period. This study aimed at investigating the factors associated with timing of first ANC visit by women in Uganda.

**Method:**

This study used secondary data from the 2016 Uganda Demographic and Health Survey (UDHS). The study population comprises of women aged 15–49 who reported to have given their last birth during the five years preceding the 2016 UDHS survey. The outcome variable for this study was the timing of first ANC visit. Univariate, bivariate, and multilevel binary logistic regression analysis was used to determine the factors associated with the utilization of timing of first ANC visit.

**Results:**

Findings show that only 30% [95%CI; 0.28–0.31] of women utilized ANC during the first trimester. Women of higher parity (4+) were less likely to utilize ANC in the first trimester compared to the lower parity (1) (AOR, 0.74, CI; 0.60–0.92). Women who reside in communities with good access to health facility were more likely to utilize ANC during the first trimester as compared to women residing in communities inaccessible to health facility (AOR, 1.36, CI; 1.04–1.77). Women who reside in less diverse ethnic communities were less likely to utilize ANC in the first trimester compared to their counterparts (AOR, 0.15, CI; 0.11–0.22).

**Conclusion:**

This study demonstrated that contextual factors are important predictors of utilization of ANC during the first trimester apart from individual, factors. It is thus important for maternal health programme interventions to consider both individual and contextual factors when encouraging women to utilize ANC services during the first trimester.

## Introduction

The timely utilization of antenatal care services is considered as one of the most important strategy for reducing maternal and infant morbidity and mortality [1, 2]. WHO in its previous focused ANC framework prescribed four ANC visits for every normal pregnancy [3], since 2016 the framework was updated from four to eight contacts, with the first contact to occur within three months (12 weeks) [4]. Early and adequate utilization of ANC from a skilled provider is associated with improved maternal health, reduced low birth weights, and lower neonatal mortality [5, 6]. Besides, timely utilization of ANC also act as a mediating factor for health facility delivery and utilization of postnatal care services [7].

In 2003, Uganda adopted the focused antenatal care framework [8] for reducing the high maternal mortality in the country. The framework prescribed that a pregnant woman must use antenatal care services at least four times, of which the first visit must occur during the first trimester [9]. However, of recent, the Ministry of Health in Uganda have updated its framework to suit the new WHO guidelines which prescribed a continuum of care for pregnant women to make eight contacts, deliver in health facility, and be attended by skilled health personnel [10, 11]. Further, the 2016 Uganda Demographic and Health Survey (UDHS) shows that 97% of women received at least one antenatal care from a skilled health personnel [12]. However, a lower proportion of these women utilized ANC services in the first trimester (30%), and maternal morbidity and mortality remained high in Uganda with little progress regarding abating maternal deaths over the past two decades [12–14].

Globally, maternal mortality has reduced by 38% [15]. Despite such an improvement, it is reported that in 2017 approximately 295,000 women died from preventable causes related to pregnancy and childbirth [15]. Over 94% of these deaths occurred in low and middle income countries, with the majority of them occurring among adolescents as compared to older women [15, 16]. It has been argued that, the high maternal mortality ratios in developing countries including sub-Saharan Africa were strongly correlated to low antenatal care service utilization [4].

Studies in the developing countries show that women initiate first antenatal visit very late after the first trimester, against the WHO recommendation [17–19]. This is also evident in sub-Saharan Africa where a low proportion (38.0%) of women utilized antenatal care services during the first trimester.

Several studies have examined the association between the individual socioeconomic and demographic characteristics and timing of antenatal service utilization [17, 20–25]. However, these studies downplay the importance of community level factors in influencing early utilization of antenatal care services [26]. These studies have concluded the important role played by individual and community factors in influencing timing of the first antenatal care visit. However, their findings varied mainly due to variation in socioeconomic context [27–32]. Moreso, several studies have been conducted on the timing of ANC in Uganda and they established the role played by socioeconomic, demographic, and cultural factors in influencing timing of ANC [7, 33–37], and these studies mainly focused on individual level factors. Besides, these studies have been inconsistent in their findings regarding the factors that influence the timing of ANC at individual-level characteristics [7, 33, 38]. Studies on context are of great importance in that, contextual-level factors reveal the characteristics of community in which the women reside and these may influence their decision towards utilization of maternal health care services, independent of individual women characteristics [39]. Uganda has an ambitious target on her vision 2040 of reducing maternal deaths to 15 deaths per 100, 000 live birth [40], also the country is a signatory to global agenda of Sustainable Development Goals (SDGs) 3.1 of reducing maternal deaths to 70 deaths per 100,000 live births by 2030 [41]. Nonetheless, studies on community level influence on timing of antenatal care service utilization in Uganda are limited. Therefore, this study aims to investigate the individual and contextual factors associated with timing of first antenatal care visit among women in Uganda. This study adds to body of knowledge by adopted DHS revised methodology which accounted for complex data at multilevel modeling by applying weights both at individual and community-level. The findings from this study would present better evidence-based results to the policymakers and other stakeholders to design and implement solutions at different levels to increase early utilization of ANC services across health facilities in Uganda.

## Methods

### Study sample

This study used secondary dataset from Uganda Demographic and Health Survey (UDHS) conducted in 2016. The study population comprises of 9443 weighted sample of women aged 15–49 who had their last birth in the five years preceding the survey and who responded to the question on when they initiated their first visit to antenatal care [12] .

### Variables and definition

#### Outcome variable

The outcome variable for this study is the timing of the first visit to the antenatal care services. The outcome variable for this study was based on WHO focused antenatal care framework [3] and 2016 Ministry of Health clinical guidelines available during the time when the survey was conducted [42]. The timing for the first antenatal care visit describes how many months of pregnancy a woman was during her first ANC visit. Timing was initially numeric ranging from 0 to 10 months and was categorized into two; first trimester (1), and not in the trimester (0) [33].

### Independent varaiables

From the 2016 Uganda Demographic and Health Surveys (UDHS) data set the following individuals and contextual level factors was run with respect to timing of first ANC visit.

### Individual-level women’s characteristics

Individual level variables include women’s level of education, employment status, place of residence, religion, parity, maternal age, marital status, household headship, family size, and wealth index. Maternal age at last birth was categorized as 15–19, 20–24, 25–29, 30–34, 35+ years. Education is defined as the highest level of education attained by the mother and categorized as: no education, primary, secondary, and above. Employment was defined as woman who reported to have worked during the past 12 months preceding the survey and was categorized as: unemployed and employed. Place of residence was defined as type of dwelling where the woman resided categorized as rural and urban. Religion is defined as the religious affiliation of the woman and was categorized as Anglican, Catholic, Muslim, and other religious groups. Parity is defined as the number of children a woman has ever given birth to and categorized as 1, 2–3, 4+ children. Marital status is defined as legally marital state categorized as single, married, living together, and previously married (separated divorced and widowed). Sex of the household head refers to male or female recognized as household head of the unit by members of the household or him/herself. Wealth index was a measure of the household socioeconomic status and was categorized in this study as poor (combined poorer and poorest quantile), middle, and rich (combined rich and richest quantile). Family size is defined as the number of household members in the household and was categorized as <= 4, 5–6 and 7+ members.

### Contextual-level characteristics

In this study, contextual or community is a sampled Enumeration Areas (EAs) (there were 696 EAs). An EA in Uganda covers an average of 130 households in each geographic area. The UDHS sampling frame for identifying Primary Sampling Units (PSUs) or EAs was based on most recent census enumeration areas created for the 2014 National Population and Housing Census (NPHS). An EA is a natural village in rural areas and a city block in urban areas [12]. There were four contextual--level variables in this study, and these include community mean distance to health facility, community socioeconomic disadvantages, community ethnicity diversity, and community media saturation. The community-level variables were constructed by aggregating individual level characteristics at the community (EA) level.

### Community mean distance to health facility

The community mean distance to health facility referred to the average distance of households to the nearest health facility in the Enumeration Areas (EAs). To generate distance variable, this study used ArcGIS Software version 10.6.1 to calculate the proximity from the Enumerated Areas (EAs) to nearest health facilities. The near function in the ArcGIS was used to locate the nearest health facility to the EA [43]. The geospatial dataset for EAs and health facility was obtained from Uganda Bureau of statistics (UBOS). The near distances from the health facilities to 685 EAs were calculated in meters and later converted into kilometres. According to World Health Organization (WHO) recommendation, a health facility must be located within an average distance of 5 km radius for easy accessibility. The values of distance generated from ArcGIS were later entered into Stata version 14.2. The distance was then recoded into dichotomous; individuals who reside within 5kms range radius were regarded to have access to health facility and those that were residing more 5 km range radius were otherwise considered to be inaccessible to health facility. Finally, the information obtained from individual variable were aggregated to EAs to form the community level variable. This variable is grouped into two outcomes, less than 5 km (accessible), and more than 5 km inaccessible.

### Community socioeconomic disadvantage

The community socioeconomic disadvantage measured the proportion of women who were socioeconomically disadvantaged. The dummy variable for DHS household wealth index was constructed from household assets and developed using principle component analysis. The PCA scores are classified as poorest, poorer, middle, richer, and richest wealth quintiles [44]. This variable was recoded into groups, poorer and the poorest formed low socioeconomic group, and the middle, rich and the richest form the high socioeconomic group. The variables were aggregated to the PSU (Enumerated Areas). The computed community-level variable was dichotomized as low and high based on the proportional value computed after checking the distribution. Since the aggregated variable is not normally distributed, a median value was used as cut-off point of categorization. Community socioeconomic status was categorised to low if the proportion of women was less than 50% and high if the proportion is above 50% [45].

### Community ethnicity diversity index

The community ethnicity diversity index is defined as the number of different ethnic groups and their proportional representation in the EA. To generate the ethnicity diversity an index formula was adopted, that captures both the number of different groups in an Enumerated Area (EA) and the relative representation of each group [46].

$$\text{Ethnicity}\;\text{diversity}\;\text{index}=1-{\textstyle\sum_{\mathrm i=1}^{\mathrm n}}\left[\frac{{\text{x}}_\text{i}}{\text{y}}\right]^2$$

Where:

x_i_ = population of ethnic group i of the EA,

y = total population of the EA, and.

n = number of ethnic groups in an EA.

The score ranges from 0 to approximately 1, the larger the index, the greater the diversity in an EA. For easy interpretation, the score is multiplied by 100; if an EA population belongs to one ethnic group, then an EA has the diversity index of 0. An EA diversity increases to 100 if the population is evenly divided into ethnic groups [39, 46, 47].

### Community media saturation

Community media saturation is defined as the proportion of women exposed to mass media information (radio, newspaper, television) in the Enumerated Areas (EAs). The responses were recoded 1, if a woman was exposed to newspaper, radio, or television and 0 if the woman was not. To create the index of exposure to media, the three forms of media were summed up of all scores for each woman, the scores ranged from 0 to 3 and the final score were recoded and categorised into two groups. A value 0 meant no access, 1 to 3 recoded 1 meant had access. This individual-level variable was aggregated to community-level variable. The computed community-level variable was dichotomized as low and high based on the proportional value computed after checking the distribution. Since the aggregated variable is not normally distributed, a median value was used as cut-off point of categorization. Community media saturation was categorised to low if the proportion of women exposure to media was less than 50% and high if the proportion is above 50% [45].

### Statistical methods

The data was analysed using Stata version 14.2 software. A bivariate analysis was performed between individual and community level characteristics and timing of first antenatal care service utilization. The chi-square test was used to examine statistical association between predictor and outcome variables. The cut-off point for level of significance was set at *p*-value less than 0.25 [45]. All variables which were found significant at bivariate model were subjected to multicollinearity test, and the test was conducted using variance inflation factor (VIF), the results shows that there is no presence of multilinearity among them (mean VIF = 1.25, Min VIF = 1.00, Max VIF = 1.95). Since the 2016 UDHS data is hierarchical in nature, therefore, multilevel binary logistic regression modelling was appropriate to test the association between individual and contextual variables and timing of first use of antenatal case service. The multilevel binary logistic regression equation of the model is stated as follows [48]:

$$\mathit{\log}\left[\frac{\pi_{ij}}{1-{\pi}_{ij}}\right]{\beta}_0+{\beta}_1{x}_{1 ij}+{\beta}_1{x}_{2 ij}+\dots +{\beta}_n{x}_{nij}+ uoj+ eij$$

Where *π*_*ij*_ is the probability of j^th^ individual woman in i^th^ community (EA) utilizing ANC in the first trimester. *(1-π*_*ij*_*)* is the probability of j^th^ individual woman in i^th^ community (EA) not utilizing ANC in the first trimester, β_0_ is the log odds of the intercept, β_1_, … β_n_ are the effect sizes of individual and community-level factors, X_1ij_... X_nij_ are independent variables of individual-level and community-level, u_Oj_ the quantities of random errors at cluster levels. e_ij_..the random error at the individual level.

Four multilevel binary logistic regression models are employed to test the association between individual and contextual variables and timing of first use of antenatal case service. In the first model, which is empty (Model 0, Null Model), no covariate was introduced. The model is used to test the random effect of between-EAs variability. The inter-class correlation coefficient (ICC) was estimated to establish if it is justified to use multilevel analysis method by showing the level of variation between-EAs. The second model (Model 1 contains only individual-level variables) determined the effects of individual-level characteristics on women’s timing of first antenatal care visit. The ICC was calculated and observed if there is any change in between-EA variability upon adding the individual-level characteristics to the empty model. The third model (Model 2 contain only the contextual-level variables) introduced community-level characteristics and excluded the individual level characteristics. In the fourth model (Model 3 contains both the individual and contextual-level variables), which is the combined model, both the individual-level and community-level characteristics were fitted to show their net fixed and random effects. The fixed-effects measures the association between individual-level and community-level factors on timing of first ANC visit was stated using the Odd Ratios (OR) with 95% confidence intervals, and *P*-values less than 5% [48, 49]. The random effect was explained using the inter-Class Correlation (I CC) using the following formula [ICC = σu^2^ / (σu^2^ + π^2^ /3)] [50]. The log likelihood ratio test was conducted to examine the adequacy of the model, and Akaike Information Criteria (AIC) was used to assess how well the different models fitted the data. To account for non-response among the sampled units in Uganda 2016 DHS data, we adopted the DHS methodology of approximating level-weights to normalize the data. The “svyset” module in the model was used to account for the complex sample by taking into consideration the three pieces of design elements; weights, EAs and the strata. Meanwhile at multivariate level, the approximated weights were applied to the model to normalize the samples for individual and community-levels (EA). This method helped to prevent the problem of inflated type one error and large confidence intervals [51].

## Results

### Univariate analysis

Table 1 shows the percentage distribution of women who had their last birth during the five years preceding the survey and had utilized ANC services by their background characteristics. A total of 9433 weighted sample of women reported to have utilized ANC during their last pregnancy preceding the 2016 UDHS. Only 29.7% (CI 0.28–0.31) of these women had their first ANC visit during their first trimester. Over half (53.6%) were aged between 20 to 29 years, and 13.7% were adolescents. The proportion of women married and living together was 81.5%. Majority of households where women resided were headed by males (73.1%). The average parity among women and average number of household members were four children per woman and six people per household correspondingly. Over half (59.8%) of the women had primary school education with slightly over a quarter (29.9%) having secondary education or higher. Majority of the women resided in rural areas (76.5%), most of the women belonged to either the Anglican or Catholic denomination (70.7%) and were employed (79.0%). Over half (41.4.%) of the women belonged to the poor wealth index bracket. Nearly three quarter (72.1%) of the women resided in communities that were inaccessible to the health facility. Over half (50.2%) of women belong to communities that were socioeconomically disadvantaged. Slightly above three quarters of the women (80.2%) belonged to a less diverse community. A higher proportion (51.5%) of women belonged to communities which are not media saturated (See Table 1).

**Table 1:** Shows the background characteristics and percentage of timing for first ANC Services utilization by selected individual and household characteristics and the level of statistical associations

Variables	Number (weighted N, %) *N* = 9443	ANC visits during the first trimester (Weighted %)	Confidence Interval (CI) 95% CI	*P-*value
**INDIVIDUAL VARIABLES**				
**Age at last birth**				0.003
< =19	1286 (13.67)	3.79	[12.86–14.52]	
20–29	5020 (53.64)	16.87	[52.44–54.85]	
30–39	2640 (27.44)	7.69	[26.38–28.53]	
40+	497 (5.25)	1.33	[4.758–5.78]	
**Marital Status**				0.006
Single	532 (5.65)	1.50	[5.076–6.289]	
Married	4032 (40.59)	12.72	[39.02–42.17]	
Living together	3735 (40.87)	11.38	[39.33–42.44]	
Previous married	1144 (12.88)	4.08	[12.03–13.79]	
**Household Head**				0.715
Male	6899 (73.06)	21.77	[71.76–74.32]	
Female	2544 (26.94)	7.91	[25.68–28.24]	
**Parity**				0.000
1	1857 (20.21)	6.15	[44.23–47.03]	
2–3	3169 (34.17)	11.04	[32.99–35.36]	
4+	4417 (45.63)	12.49	[44.23–47.03]	
**Family Size**				0.000
< =4	3073 (33.54)	10.80	[32.21–34.9]	
5–6	2941 (30.93)	9.28	[29.91–31.97]	
7+	3429 (35.53)	9.60	[34.21–36.86]	
**Level of Education**				0.031
No education	1176 (10.31)	3.44	[9.395–11.31]	
Primary	5764 (59.78)	17.16	[58.09–61.44]	
Secondary or Higher	2503 (29.91)	9.08	[28.17–31.7]	
**Place of Residence**				0.760
Urban	1969 (23.53)	7.07	[74.79–78.07]	
Rural	7474 (76.47)	22.61	[21.93–25.21]	
**Wealth Index**				0.733
Poor	4412 (41.19)	12.02	[39.12–43.3]	
Middle	1752 (18.96)	5.67	[17.78–20.18]	
Rich	3279 (39.85)	11.99	[37.49–42.26]	
**Religion**				0.084
Anglican	2981 (31.11)	9.20	[29.58–32.68]	
Catholic	3931 (39.56)	12.30	[37.66–41.49]	
Muslim	1085 (13.81)	3.92	[12.32–15.44]	
Other’s	1446 (15.52)	4.45	[14.31–16.82]	
**Employment Status**				0.116
Unemployed	1877 (21)	5.85	[19.76–22.29]	
Employed	7566 (79)	23.83	[77.71–80.24]	
**COMMUNITY VARIABLES**				
**Community Distance to Health Facility**				0.074
Inaccessible	6540 (72.59)	16.95	[71.03–74.1]	
Accessible	2758 (27.41)	12.95	[25.9–28.97]	
**Community Socioeconomic Status**				0.329
Low	5400 (50.24)	15.24	[46.68–53.79]	
High	4043 (49.76)	14.44	[46.21–53.32]	
**Community Ethnicity Diversity Index**				0.000
Less Diverse	7473 (80.21)	16.52	[78.76–81.59]	
More Diverse	1970 (19.79)	13.16	[18.41–21.24]	
**Community Media Saturation**				0.112
Less Saturated	5235 (51.47)	15.82	[47.79–55.14]	
Saturated	4208 (48.53)	13.86	[44.86–52.21]	

### Prevalence of timing of first ANC visit across the explanatory variables

Table 1 shows the percentage distribution of women who had their last birth 5 years prior the 2016 UDHS and utilized ANC services by timing of ANC and background characteristic. Close to about a third of the women who utilized ANC services (30, 95%CI; 0.28–0.31) did so during the first trimester. The proportion of women who first utilized ANC services during the first trimester differed significantly by woman’s age at birth of last child, marital status, woman’s parity, household size, education, Community Ethnicity diversity index.

Over one eighth (16.7%) of women aged 20 to 29 years old had their first ANC visit during their first trimester as compared to women aged less than 19 years old (3.8%), women aged 30 to 39 years (7.7%) and those aged 40 years and above (26%). A third (32.5%) of the women were previously married utilized ANC services during the first trimester compared to those married (31.4%), and those living together and single (1.3%) respectively. A higher proportion (12.49%) of women who had four or more children made their first ANC visit compared to their counterparts with 2 to 3 children (11.04%) and 1 child (6.15%). Only 9.6%of women who belong to households with seven or more members first utilized ANC services during their first trimester as compared to about a third (9.3%) and 10.8% who resided in households with fours or less members and 5 to 6 members respectively. Over one eighth (17.16%) of women with primary education utilized ANC services during the first trimester as compared to those with no education and secondary or higher level of education (3.4 and 9.08% respectively). The proportion of women who utilized ANC services in the first trimester was significantly higher (26.93%) for women residing in from communities which are not ethnically diverse compared to their counterparts who resided communities which are not ethnically diverse (2.8%) (See Table 1).

### Measure of association (fixed effects) results

Model 4 in Table 2 contains individual and community level variables. In this model, timing of first ANC utilization was association with partially with marital status, parity, community distance to health facility, and community ethnicity diversity. The odds of utilizing ANC services in the first trimester remained higher among formerly married (AOR, 1.35, CI; 0.99–1.83) compared to the never married (single). Women who had four or more children were less likely to utilize ANC during the first trimester compared to those who had only a child (AOR, 0.74, CI; 0.60–0.92). Further, women who lived in communities that were accessible to the nearest health facility were almost three times more likely to attend ANC during the first trimester, compared to those who resided in Inaccessible communities (AOR, 1.36, CI; 1.04–1.77). Women who lived in ethnic diverse communities were less likely to utilize ANC in the first trimester, compared to their counterpart (AOR, 0.15, CI; 0.11–0.22).

**Table 2:** Multilevel Analysis of Odd Ratios on the effect of Individual, Household and Community level factors in influencing the timing of ANC Utilization in Uganda, UDHS 2016

Variables	Model 1	Model 2	Model 3	Model 4
	Empty Model	Individual level	Community level	Individual/Community level
	Odd Ratio (95% CI)	Odd Ratio (95% CI)	Odd Ratio (95% CI)	AOR (95% CI)
**INDIVIDUAL VARIABLES**				
**Age at last birth**				
< =19		1.00		1.00
20–29		1.18 (0. .97–1.44)		1.18 (0.97–1.44)
30–39		1.12 (0.89–1.42)		+ 1.14 (0.89–1.46)
40+		1.05 (0.70–1.41)		1.05 (0.73–1.51)
**Marital Status**				
Single		1.00		1.00
Married		1.13* (1.01–1.70)		1.25 (0.95–1.64)
Living together		1.11 (0.86–1.44)		1.05 (0.81–1.37)
Previous married		1.41* (1.05–1.89)		1.35 (0.99–1.83)
**Parity**				
1		1		1.00
2–3		0.93 (0.78–1.11)		0.89 (0.75–1.07)
4+		0.78* (0.63–0.96)		0.74* (0.60–0.92)
**Family Size**				
< =4		1.00		1.00
5–6		0. .94 (0.78–1.11)		0.95 (0.81–1.12)
7+		0.87 (0.63–0.96)		0.85 (0.72–1.01)
**Level of Education**				
No education		1.00		
Primary		0.93 (0.77–1.13)		
Secondary or Higher		1.001 (0.80–1.26)		
**Religion**				
Anglican		1.00		1.00
Catholic		0.95 (0.83–1.09)		0.99 (0.86–1.15)
Muslim		0.84 (0.68–1.05)		0.89 (0.71–1.11)
Other’s		0.97 (0.79–1.13)		0.96 (0.80–1.15)
**Employment Status**				
Unemployed		1.00		1.00
Employed		1.16 (0.99–1.35)		1.17 (0.99–1.37)
**COMMUNITY VARIABLES**				
**Community Distance to Health Facility**				
Inaccessible			1.00	1
Accessible			1.35**(1.04–1.75)	1.36*(1.04–1.77)
**Community Ethnicity Diversity Index**				
Less Diverse			1.00	1.00
More Diverse			0.15***(0.11–0.22)	0.15***(0.11–0.22)
**Community Media Saturation**				
Less Saturated			1.00	
Saturated			0.89 (0.71–1.13)	
Random effect results:				
PSU Variance (95% CI)	0.757(0.62–.93)	0.734(0.60–0.90)	0.987(0.793–1.229)	0.965(0.78–1.20)
ICC	0.19	0.18	0.23	0.23
Wild chi-square and p-value	Ref	χ^2^ = 37.73, *p* < 0.003	χ^2^ = 116.70, *p* < 0.000	χ^2^ = 159.31, p < 0.000
Model fitness:				
Log-likelihood	−2,041,788.8	−2,031,440.7	−1,894,570.9	−1,885,488
AIC	4,083,582	4,062,917	3,789,152	3,771,012
PSU	649	649	649	649
N	9443	9443	9298	9298

The Empty Model contain no variables, but it partitioned the variance into two component parts. *AIC* Akaike Information Criterion, *CI* Confidence Interval,® Reference category,® reference category, *AOR* Adjusted Odd Ratio’s, *ICC* Inter-classical correlations, Significant level, ***p = <.001, **p < .01, *p < .05. *PSU* Primary sampling unit, *N* Number

### Measure of variations (random effects) results

The random intercept of Model 1 shows that utilization of ANC in the first trimester was statistically significant across the Enumerated Areas (EA)(*τ* = 0.76, 95%CI; (0.62–.93). The model 1 revealed that 19% of the variation in the utilization of ANC in the first trimester was associated to the between-EA variation (ICC = 0.19). The between-EA difference decreased from 19% in Model 1 to 18.0% that had only the individual and community level factors (Model 2). The between-EA difference increased from 18% Model 2 to 23% in the community-level only (3). Lastly, ICC decreased to 22% in the complete model that contained both the individual and community-level factors. This means that the differences in the probability of utilization of timing of ANC in the first trimester can be explained by the variances across the EAs. The overall model fit statistics AIC shows a successive decrease, which shows that there is considerable improvement from the empty model to the final model. This confirms the goodness of fit of final model established in the analysis. Therefore, Model 4 was chosen for forecasting the utilization of ANC in the first trimester among women in Uganda (See Table 2).

## Discussion

This study examined the factors associated with the utilization of timing of first ANC visit.

A significant majority of women in Uganda begin to utilize antenatal care services late. There is a low proportion of women who utilized antenatal care services during the first trimester. These findings were in line with evidence from other developing countries, especially in sub-Saharan Africa which indicate that a small proportion of women reported early to utilize antenatal care services [17, 18, 36, 52, 53]. This study revealed that both the individual characteristics and community context were important predictors of timing of first utilization of antenatal care services in Uganda.

Findings in this study indicate that individual level factors linked with use of antenatal care services during the first trimester included marital status, and parity. In Uganda, women who were formerly married were more likely to utilize ANC services in the first trimester compared to other marital status. Literature from studies conducted in Zambia and Tanzania show that women’s marital status was a significant predictor of early use of ANC services [17, 20]. These could be attributed to the level of autonomy among women. Ugandan communities are mainly dominated by patriarchal structure where power in the household mainly are vested on men, hence this has a great effect on gender relations [21, 22].

Women of higher parity are less likely to utilize antenatal care in the first trimester compared to those with lower parity. The main reasons for late attendant of antenatal care could be attributed to poor attitudes towards ANC and previous pregnancy experiences regarding ANC attendance. For instance, a study was conducted in Mulago hospital which is the main referral hospital in Uganda, the reasons women gave for late ANC attendance were, “I was busy”, “I was lazy”, “I got tired of attending antenatal care in previous pregnancies”, “I have had children before [54].

Community contextual factors associated with timing of antenatal care service utilization included the community mean distance to the nearest health facility, community ethnicity diversity. In this study, the community mean distance to health facility was negatively associated with timing of first visit to antenatal care service utilization among women in Uganda. Women who reside in communities that were averagely inaccessible to health facility were less likely to initiate their first visit to antenatal care service utilization during the first trimester compared to those residing in communities considered accessible. Contrary to these findings, findings from studies in Zambia and Benin showed no association between timing of first visit to antenatal care service utilization and distance to health facility [55, 56]. Studies in sub–Saharan Africa opined distance as a factor in the utilization of health facilities [44, 57–59]. For instances, Gabrysch and Campbell [58] found distance to health facility having two effects; distance as disincentive to motivate people from seeking care, and an obstacle for reaching health facilities, and is mainly augmented by poor transport and lack of roads.

Community ethnicity diversity was associated with timing of antenatal care services. Women who reside in diverse community were less likely to utilize ANC in the first trimester, compared to their counterparts. The main reason associated to this could be that communities which are homogenous share common norms, which enables cooperation among co-ethnics in terms of information sharing could be easier compared to heterogenous communities. The findings from this study is in line with other studies in Uganda and elsewhere which established an association between ethnicity diversity and healthcare seeking behaviour [39, 60].

### Limitation and strength of the study

This study has its own limitations and the first there is and possibility of recall bias which may occur due to the retrospective nature of the study whose answers may not be verified due to the secondary nature of the data. The other limitations could be associated with community-level factors which were aggregated from individual-level information, these could lead to ecological fallacy. Despite these limitations, this study used the recent multilevel methodology suggested by DHS which involves weight approximation, this has contributed greatly to the body of knowledge regarding multilevel analysis. The outcome variable can be easily remembered as it relates to whether a woman utilized ANC during the first three months or later. This study contributed to a body of knowledge by examining the effects of community level variables together with individual level variables, on the timing of antenatal care utilization. Such studies are relatively scarce in Uganda. One of the main strengths of this study is that this study measured distance to health facility using ArcGIS software on 685 out of 696 enumeration areas whose coordinates were found to be valid from each EA to the nearest health facility [47]. This helped to eliminate distance bias created by determining accessibility by using perceived distance or perceived accessibility as a proxy. The study also adopted the Simpson diversity index [46] to measure the influence of ethnicity in the utilization of antenatal care which is relatively scarce in the context of Uganda. The study used women from enumerated areas as unit of analysis, such information can be generalized to the study population.

## Conclusion

This study managed to demonstrate the importance of community context and individual level factors influencing timing of ANC. The contextual factors associated with the timing of first ANC visit were community mean distance to health facility, community ethnicity diversity. In Uganda, women residing in communities that were not accessible to the nearest health facility were less likely to utilize ANC services during their first trimester. Communities which are ethnically diverse were more likely to utilize ANC services during their first trimester. Individual level factors associated with the timing of first ANC included marital status, and parity. Policy interventions geared towards improving the quality of maternal and child health services in the nearest health facilities are recommended. Health education programs which target women of higher parity to encourage them to attend ANC in the first trimester is encourage. This study is mainly quantitative, there is need to combine qualitative and quantitative study to understand the root cause of late utilization of ANC.

## Data Availability

The dataset can be accessed through this website; https://dhsprogram.com/data/dataset_admin/login_main.cfm?CFID=6055127&CFTOKEN=416a39e1e52181a9-CCE2DAA5-A212-565C-40BD6F8E8C8E5041. Registration is required. This study used the UGIR70FL (Individual Recode –Women with completed interviews – Uganda, 2016).

## Abbreviations

ANC: Antenatal Care
EA: Enumerated Areas
ICC: inter-class correlation
OR: odd ratio PNC Postnatal Care
PVC: Proportional Variance Change
UDHS: Uganda Demographic and Health
WHO: World Health Organization
AOR: Adjusted Odd Ratios

## References

[1] Fagbamigbe AF, Idemudia ES (2017). Wealth and antenatal care utilization in Nigeria: policy implications. Health Care for Women International.

[2] Gulema H, Berhane Y (2017). Timing of first antenatal care visit and its associated factors among pregnant women attending public health facilities in Addis Ababa, Ethiopia. Ethiop J Health Sci.

[3] World Health Organization (2002). WHO antenatal care randomized trial: manual for the implementation of the new model.

[4] World Health Organization (2016). WHO recommendations on antenatal care for a positive pregnancy experience.

[5] Cokkinides V (2001). Health insurance coverage-enrollment and adequacy of prenatal care utilization. J Health Care Poor Underserved.

[6] Joshi C, Torvaldsen S, Hodgson R, Hayen A (2014). Factors associated with the use and quality of antenatal care in Nepal: a population-based study using the demographic and health survey data. BMC Pregnancy Childbirth.

[7] Atuhaire R, Atuhaire LK, Wamala R, Nansubuga E (2020). Interrelationships between early antenatal care, health facility delivery and early postnatal care among women in Uganda: a structural equation analysis. Glob Health Action.

[8] Nabwire J (2017). Assessing the implementation of focused antenatal care and factors influencing its implementation across health facilities in Jinja District, Uganda.

[9] Lincetto O, Mothebesoane-Anoh S, Gomez P, Munjanja S. Antenatal care. Opportunities for Africa's newborns: Practical data, policy and programmatic support for newborn care in Africa. 2006:55–62. Accessed from https://scholar.google.com/scholar?hl=en&as_sdt=0%2C5&q=Lincetto+O%2C+Mothebesoane-Anoh+S%2C+Gomez+P%2C+Munjanja+S%3A+Antenatal+care.+Opportunities+for+Africa%27s+newborns%3A+Practical+data%2C+policy+and+programmatic+support+for+newborn+care+in+Africa+2006%3A55-62.&btnG=

[10] Muwema M, Kaye DK, Edwards G, Nalwadda G, Nangendo J, Okiring J, Mwanja W, Ekong EN, Kalyango JN, Nankabirwa JI (2022). Perinatal care in Western Uganda: prevalence and factors associated with appropriate care among women attending three district hospitals. PLoS One.

[11] Ministry of Health (2022). Essential Maternal and Newborn Clinical Care Guidelines for Uganda.

[12] UBOS (2018). & ICF: Uganda demographic and health survey 2016. In.

[13] UBOS & ICF (2006). Uganda demographic and health survey.

[14] UBOS & ICF (2012). Uganda demographic and health survey 2011. In.

[15] Maternal mortality: Trends in estimates of maternal mortality ratio (maternal deaths per 100,000 live births) 2000-2017 [https://data.unicef.org/topic/maternal-health/maternal-mortality/].

[16] Maternal mortality: WHO. World Health Organization [https://www.who.int/news-room/fact-sheets/detail/maternal-mortality].

[17] Chewe MM, Muleya MC, Maimbolwa M (2016). Factors associated with late antenatal care booking among pregnant women in Ndola District, Zambia. Afr J Midwifery Womens Health.

[18] Fulpagare PH, Saraswat A, Dinachandra K, Surani N, Parhi RN, Bhattacharjee S, Purty A, Mohapatra B, Kejrewal N, Agrawal N (2019). Antenatal care service utilization among adolescent pregnant women–evidence from Swabhimaan programme in India. Front Public Health.

[19] Gross K, Alba S, Glass TR, Schellenberg JA, Obrist B (2012). Timing of antenatal care for adolescent and adult pregnant women in South-Eastern Tanzania. BMC Pregnancy Childbirth.

[20] Njiku F, Wella H, Sariah A, Protas J. Prevalence and factors associated with late antenatal care visit among pregnant women in Lushoto, Tanzania. Tanzania J of Health Res. 2017;19(3).

[21] Gopal P, Fisher D, Seruwagi G, Taddese HB (2020). Male involvement in reproductive, maternal, newborn, and child health: evaluating gaps between policy and practice in Uganda. Reprod Health.

[22] Kuuire VZ, Kangmennaang J, Atuoye KN, Antabe R, Boamah SA, Vercillo S, Amoyaw JA, Luginaah I (2017). Timing and utilisation of antenatal care service in Nigeria and Malawi. Global Public Health.

[23] Efendi F, Chen C-M, Kurniati A, Berliana SM (2017). Determinants of utilization of antenatal care services among adolescent girls and young women in Indonesia. Women & health.

[24] Barman B, Saha J, Chouhan P (2020). Impact of education on the utilization of maternal health care services: an investigation from National Family Health Survey (2015–16) in India. Child Youth Serv Rev.

[25] Dewau R, Muche A, Fentaw Z, Yalew M, Bitew G, Amsalu ET, Arefaynie M, Mekonen AM (2021). Time to initiation of antenatal care and its predictors among pregnant women in Ethiopia: cox-gamma shared frailty model. PLoS One.

[26] Matthews SA, Bina G. Contextual influences on the use of antenatal care in Nepal. DHS Geographic Studies 2. Calverton, Maryland USA: ORC Macro; 2004.

[27] Teshale AB, Tesema GA (2020). Prevalence and associated factors of delayed first antenatal care booking among reproductive age women in Ethiopia; a multilevel analysis of EDHS 2016 data. PLoS One.

[28] Belay DG, Aragaw FM, Anley DT, Tegegne YS, Gelaye KA, Tessema ZT (2021). Spatiotemporal distribution and determinants of delayed first antenatal care visit among reproductive age women in Ethiopia: a spatial and multilevel analysis. BMC Public Health.

[29] Chama-Chiliba CM, Koch SF (2015). Utilization of focused antenatal care in Zambia: examining individual-and community-level factors using a multilevel analysis. Health Policy Plan.

[30] Magadi MA, Madise NJ, Rodrigues RN (2000). Frequency and timing of antenatal care in Kenya: explaining the variations between women of different communities. Soc Sci Med.

[31] Alem AZ, Yeshaw Y, Liyew AM, Tesema GA, Alamneh TS, Worku MG, Teshale AB, Tessema ZT (2022). Timely initiation of antenatal care and its associated factors among pregnant women in sub-Saharan Africa: a multicountry analysis of demographic and health surveys. PLoS One.

[32] Boco GA (2010). Individual and community level effects on child mortality: an analysis of 28 demographic and health surveys in sub-Saharan Africa.

[33] Sserwanja Q, Nabbuye R, Kawuki J (2022). Dimensions of women empowerment on access to antenatal care in Uganda: a further analysis of the Uganda demographic health survey 2016. Int J Health Plann Manag.

[34] Kawungezi PC, AkiiBua D, Aleni C, Chitayi M, Niwaha A, Kazibwe A, Sunya E, Mumbere EW, Mutesi C, Tukei C (2015). Attendance and utilization of antenatal care (ANC) services: multi-center study in upcountry areas of Uganda. Open J Prev Med.

[35] Agaba P, Magadi M, Onukwugha F, Misinde C (2021). Factors associated with the timing and number of antenatal care visits among unmarried compared to married youth in Uganda between 2006 and 2016. Soc Sci.

[36] Turyasiima M, Tugume R, Openy A, Ahairwomugisha E, Opio R, Ntunguka M, Mahulo N, Akera P, Odongo-Aginya E (2014). Determinants of first antenatal care visit by pregnant women at community based education, research and service sites in northern Uganda. East Afr Med J.

[37] Bbaale E (2011). Factors influencing timing and frequency of antenatal care in Uganda. Australas Med J.

[38] Sserwanja Q, Mutisya LM, Musaba MW (2022). Exposure to different types of mass media and timing of antenatal care initiation: insights from the 2016 Uganda demographic and health survey. BMC Womens Health.

[39] Achia TN, Mageto LE (2015). Individual and contextual determinants of adequate maternal health care services in Kenya. Women & health.

[40] Government of Uganda (2013). Uganda Vision 2040.

[41] UN (United Nations). Resolution Adopted by the General Assembly on 25 September 2015 70/1. Transforming Our World: The 2030 Agenda for Sustainable Development

[42] Ministry of Health (2016). Uganda Clinical Guidelines 2016: National Guidelines for Management of Common Conditions.

[43] Nigatu AM, Gelaye KA, Degefie DT, Birhanu AY (2019). Spatial variations of women’s home delivery after antenatal care visits at lay Gayint District, Northwest Ethiopia. BMC Public Health.

[44] Ononokpono DN, Odimegwu CO, Imasiku E, Adedini S (2013). Contextual determinants of maternal health care service utilization in Nigeria. Women & health.

[45] Abate MG, Tareke AA (2019). Individual and community level associates of contraceptive use in Ethiopia: a multilevel mixed effects analysis. Arch of Pub Health.

[46] Simpson EH (1949). Measurement of diversity. Nature.

[47] Uthman OA (2010). Does it really matter where you live? A multilevel analysis of social disorganization and risky sexual behaviours in sub-Saharan Africa. DHS Working Papers.

[48] Yebyo HG, Gebreselassie MA, Kahsay AB (2014). Individual and community-level predictors of home delivery in Ethiopia: a multilevel mixed-effects analysis of the 2011 Ethiopia National Demographic and health survey.

[49] Ejembi CL, Dahiru T, Aliyu AA. Contextual factors influencing modern contraceptive use in Nigeria. DHS Working Papers. 2015;120.

[50] Gulliford M, Adams G, Ukoumunne O, Latinovic R, Chinn S, Campbell M (2005). Intraclass correlation coefficient and outcome prevalence are associated in clustered binary data. J Clin Epidemiol.

[51] Elkasabi M, Ren R, Pullum TW. Multilevel modeling using DHS Surveys: A framework to approximate level-weights (DHS Methodological Reports No. 27). 2020, Article DHS Methodological Reports No. 27.

[52] Gross K, Alba S, Glass TR, Schellenberg JA, Obrist B (2012). Timing of antenatal care for adolescent and adult pregnant women in South-Eastern Tanzania. BMC Pregnancy Childbirth.

[53] Tariku A, Melkamu Y, Kebede Z. Previous utilization of service does not improve timely booking in antenatal care: cross sectional study on timing of antenatal care booking at public health facilities in Addis Ababa. Ethiop J Health Dev. 2010;24(3).

[54] Kisuule I, Kaye DK, Najjuka F, Ssematimba SK, Arinda A, Nakitende G, Otim L (2013). Timing and reasons for coming late for the first antenatal care visit by pregnant women at Mulago hospital, Kampala Uganda. BMC Pregnancy Childbirth.

[55] Tanou M, Kishida T, Kamiya Y. Geographical access to health facilities and maternal healthcare utilization in Benin: a cross-sectional study. Research Square. 2021. Accessed at https://assets.researchsquare.com/files/rs-406731/v1/34846a00-0d83-479e-a335-2427d4301c18.pdf?c=1631881424.

[56] Kyei NN, Campbell OM, Gabrysch S (2012). The influence of distance and level of service provision on antenatal care use in rural Zambia. PLoS One.

[57] Onasoga OA, Afolayan JA, Oladimeij BD (2012). Factor’s influencing utilization of antenatal care services among pregnant women in Ife central LGA, Osun state Nigeria. Adv Appl Sci Res.

[58] Gabrysch S (2010). The influences of distance on health facility delivery in rural Zambia.

[59] Gabrysch S, Campbell OM (2009). Still too far to walk: literature review of the determinants of delivery service use. BMC Pregnancy Childbirth.

[60] Björkman M, Svensson J (2010). When is community-based monitoring effective? Evidence from a randomized experiment in primary health in Uganda. J Eur Econ Assoc.

[61] Nankinga O, Aguta D, Kabahuma C. Trends and determinants of anemia in Uganda: further analysis of the demographic and health surveys. DHS Working Papers. 2019;149. 10.1186/s12889-019-8114-1 PMC6937990 31888579

